# Non-Aqueous Electromigration Analysis of Some Degradation Products of Carvedilol

**Published:** 2014

**Authors:** Abolghasem Jouyban, Mohammad Hasanzadeh, Nasrin Shadjou

**Affiliations:** a *Drug Applied Research Center and Faculty of Pharmacy, Tabriz University of Medical Sciences, Tabriz 51664, Iran.*; b *Liver and Gastrointestinal Diseases Research Center, Tabriz University of Medical Sciences, Tabriz 51664, Iran. *; c *Biochemistry Lab, Pasteur Institute of Iran, Tehran, Iran.*

**Keywords:** Capillary electrophoresis, Carvedilol, Photo and force degradations, Non-aqueous

## Abstract

A capillary electrophoresis method was used for assay of some degradation products of carvedilol. The optimized parameters were as; running buffer 80 mM acetate dissolved in methanol/ethanol mixture (65:35% v/v), applied voltage of 19 kV, temperature is 20 ºC and the wavelength range of 200-350 nm. The results indicate that the proposed capillary electrophoresis method could effectively separate carvedilol from its degradation products and can be employed as a stability indicating assay method. In addition, the presence of a new unknown degradation product was discovered by this method. In addition, capillary electrophoresis behaviour of carvedilol in photo/force degradation conditions gave valuable information concerning the dissimilarities of their ionization. Results indicated that the capillary electrophoresis proposed method can be used for the determination of carvedilol in human serum. Finally, accuracy of the proposed method was established by recovery experiments from spiked human serum samples.

## Introduction

Nowadays, growing emphasis is placed on testing of purity and stability of active substances especially to assure the high quality of drug products in connection with safe medical care. Impurity is described as any component of the drug substance or drug product that is not the chemical entity defined as the drug substance or other additives to the drug product. This term is broad enough to include degradation products as impurities (1). In ICH guidelines, the degradation product is defined as an impurity resulting from a chemical change in the drug substance during manufacture and/or storage of the drug product (2). 

Degradation products are often present in very low levels. It is necessary not only to identify degradation products but also to determine their amount present in drug substances and drug products. Capillary electrophoresis (CE), high performance liquid chromatography (HPLC) and gas chromatography (GC) techniques are the widely used methods in 7^th^ Edition of European pharmacopoeia and in USP 28 for the assay of bulk drug materials (3, 4). Such methods enable to perform analyses with high specificity, sensitivity, sufficient precision, and reproducibility. Because of different analytical mechanisms of CE and HPLC, these methods provide orthogonal techniques to follow the degradation products of drugs. The advantage of CE method is the possibility to get qualitative and quantitative information about component content in the sample during one-step analysis. As an example, it could be easily coupled with a mass spectrometer which facilitates the structure elucidation of the degradation products.

Carvedilol (CVD; 1-(carbazol-4-yloxy-3- [[2-(O-methoxyphenoxy)ethyl]amino]-2 propanol) is used for treatment of mild to moderate hypertension, angina pectoris, and congestive heart failure. Several chromatographic methods have been developed for analysis of CVD in biological fluids such as whole blood, plasma, and serum (5-17). Most methods have focused on separation of the enantiomers (8-12) and no comprehensive information on stability-indicating analysis of CVD is available in the literature. The available stability indicating methods include a UV spectroscopic method of Imran *et al.* (18) and a LC method of Rizwan *et al.* (19) for the analysis of CVD degradation and study of kinetic degradation of CVD. 

Stability testing of an active substance or finished product provide evidence on how the quality of a drug substance or drug product varies with time influenced by a variety of environmental factors such as pH, temperature and light. Knowledge from stability studies enables us to understand the long-term effects of the environment on the drugs. Stability testing provides information about degradation mechanisms, potential degradation products, possible degradation pathways of drug as well as interaction between the drug and the excipients in the drug formulation. Since daylight-induced degradation of the drug may have a negative impact on the quality, safety and effectiveness of pharmacotherapy, research focused on this point is definitely reasonable. The objective of this work (at continuing our study on beta-blockers and impurity analysis (20, 21) is to use of a CE method for degradation study of CVD. 

## Experimental

*Reagents and chemicals*

CVD was supplied by Daru Pakhsh Pharmaceutical Co. (Tehran, Iran). Methanol, ethanol, glacial acetic acid, sodium acetate, sodium hydroxide were purchased from Merck (Darmstadt, Germany). De-ionized water was used for preparing the acetate buffer. Stock solution (1.14 mg/mL) was prepared by dissolving CVD in 25 mL running buffer.

*Apparatus*

The electrophoresis analyses were performed using an Agilent 7100 capillary electrophoresis (Germany) driven with Agilent Chem. Station software. A fused silica capillary (57 cm length and 75 µm I.D.) was used for electrophoresis experiments. The background electrolyte consisted of acetate buffer (80 mM) dissolved in methanol/ethanol mixture (65:35%). Samples were injected into the capillary by pressure 50 mbar, for 6 s. The applied voltage was 15-30 kV range and the optimum voltage obtained as 19 kV. The capillary was washed between runs with a sequence of rinses: 0.1 M sodium hydroxide (1 min), water (0.5 min), 0.1 M hydrochloric acid (0.5 min), and water (0.5 min), followed by running buffer for 3 min to ensure reproducibility of the assay. Buffer ionic strength and pH as well as applied voltage were optimized. Detection was performed by a UV-DAD detector at 200-350 nm range.

## Results and Discussion

*Photodegradation of CVD*

The most obvious result of drug photodegradation is a loss of potency of the product. The drug substance can also cause light-induced side effects after administration to the patient by interaction with endogenous substances. Therefore, two aspects of drug photostability must be considered: *in-vitro* and *in-vivo* stability. Despite of concern about *in-vitro* stability or *in*-*vivo* effects, characterization of the photochemical properties of drug substances and drug formulations is a part of the formulation work and cannot be ignored. Many drug substances and drug products are found to decompose *in-vitro *under exposure to light, but the practical consequences will not necessarily be the same in all cases. This emphasizes the importance of study of reaction mechanisms and sensitizing properties of CVD and its degradation products in the evaluation of photostability of drugs.

For monitoring the stability of CVD by CE, (1.14 mg) of powder distributed in a thin layer (<1 mm), was exposed to continuous daylight for 4 weeks at room temperature (25 °C). The other sample was kept in a dark place to compare degradation process with and without daylight exposure.

**Figure 1:**
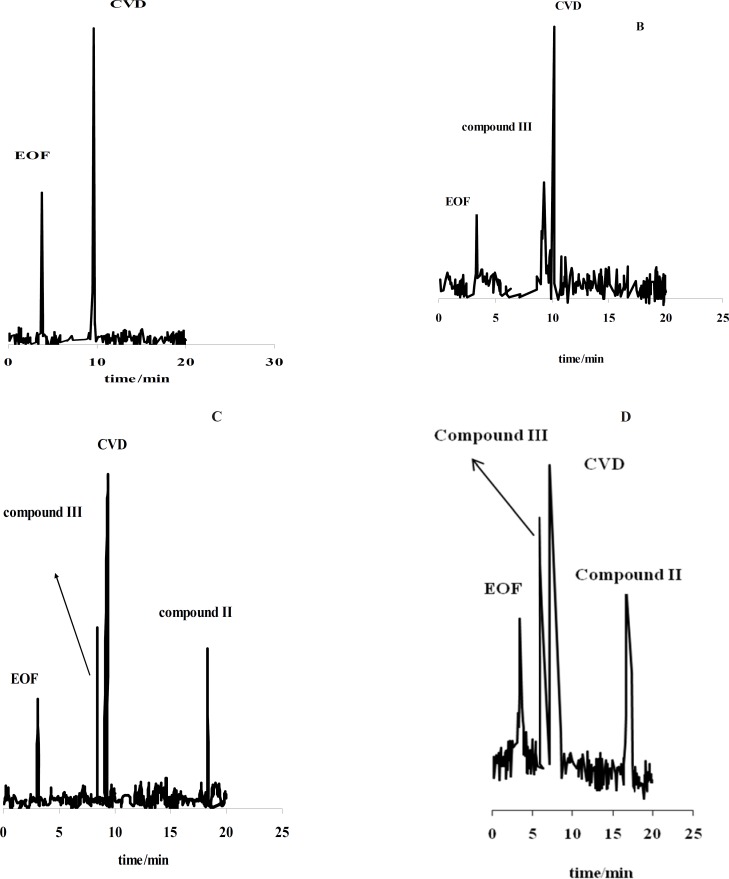
Electrophorogram of 1.14 mg/mL CVD for time=0 (immediately after preparation of CVD) (**A**) and (B-D) after 10-30 days of exposure to daylight. Acetate buffer in methanol: ethanol (65:35 v/v) with the apparent pH of 4.1.

Figure 1(A-D) shows the effect of the exposure time on degradation products of CVD separated by the proposed CE method which time is 0 (immediately after preparation of CVD) up to 30 days. It is observed when time=0, only one peak was observed (migration time= 9.63 min) that related to CVD detection (Figure 1A). The presence of the degradation products were observed every week for 4 weeks. The first degradation product (migration time=9.33 min) was detected in the exposed sample by electrophoresis analysis in the second week (exactly 10 days) (Figure 1B). Figures 1C and 1D show the electrophorogram of a sample after 3 and 4 weeks of exposure to daylight. Two peaks with migration times of 8.69 and 16.70 min were observed. Figures 1C and D indicated that the first time for detection of degradation product II is 21 days.

*Study of photodegradation mechanism*

The photoassay should lead to determination of degradation pathways; identification of degradation products; and evaluation of sensitizing properties of degradation products. Sunlight has a very high output in the visible (400 to 800 nm) and infrared (800 to 3200 nm) regions. The only importance that infrared radiation can accrue in the context of photodegradation is that the sample can be heated, thereby activating thermal decomposition. The result can be decomposition of the drug substance or destruction of the formulation, unless scavengers of free radicals are added to protect the preparation.

**Scheme 1:**
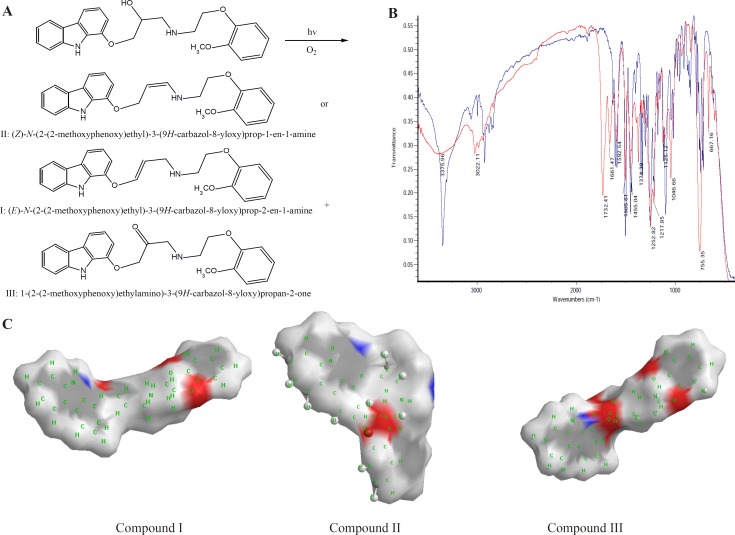
A) Degrading procedure of CVD on photodegradation conditions B) FTIR spectrum of CVD at t=0 and t=19 day of exposition to daylight. C) Partial surface area of CVD photodegradation products

CVD can be exposed to daylight to obtain its decomposition products. As the first degradation product, 1-(2-(2-methoxyphenoxy) ethylamino)-3-(9H-carbazol-8-yloxy) propan-2-one (compound III of Table 1) was found by electrophoresis method [22, 23]. From electrophoresis results, it could be suggested that compound III is the first degradation product of CVD while one of two forms of (E)-N-(2-(2-methoxyphenoxy) ethyl)-3-(9H-carbazol-8-yloxy)prop-2-en-1-amine (compound I of Table 1) or (Z)-N-(2-(2-methoxyphenoxy)ethyl)-3-(9H-carbazol-8-yloxy) prop-1-en-1-amine (compound II of Table 1), is the second product which is further investigated in this study. In the oxidation of CVD, compound II is an intermediate. The degradation products such as compound I can also be formed by oxidative degradation of the intermediate. The chemical structures of CVD and its photodegradation products are given in Scheme 1A. Degradation product III is a Pharmacopoeia impurity and compound I is a non-Pharmacopoeia impurity.

**Table 1:** Information of CVD degradation product

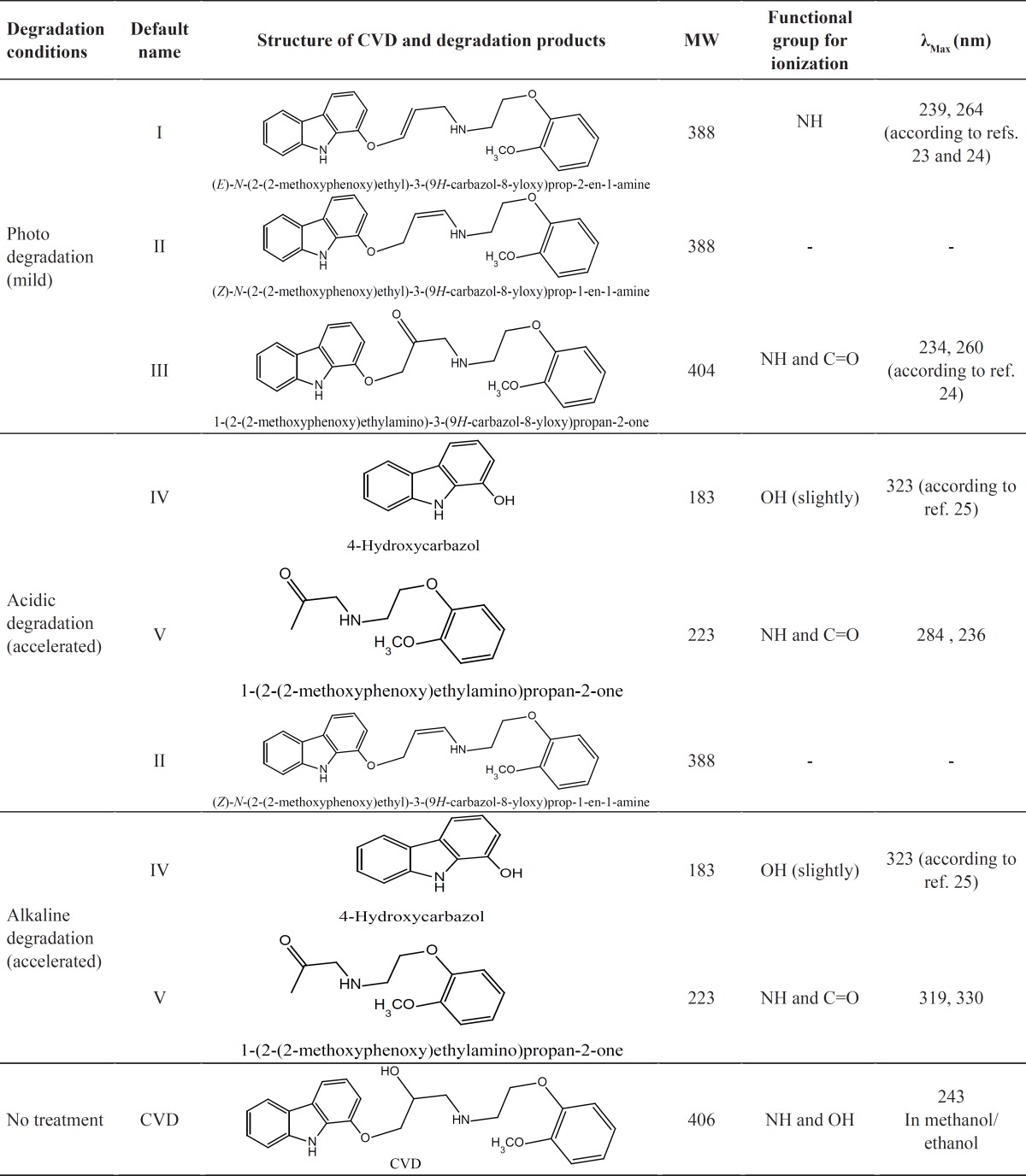

**Figure 2:**
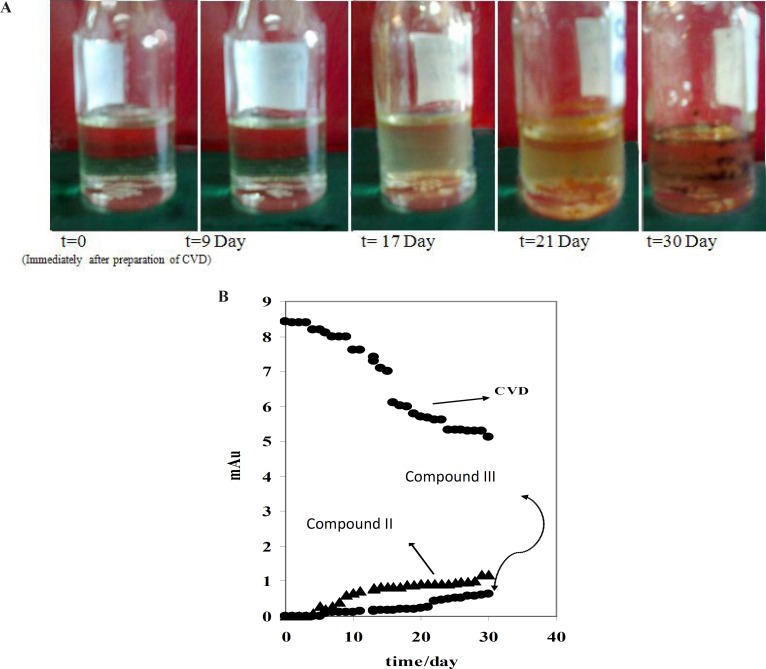
A) CVD discolor *vs.* time of exposition. B) Variation of mAU *vs.* time of daylight exposure for CVD and detected degradation products

Figure 1 (B-D) indicated that compound III with the migration time of 9.33 min is the first product of degradation pathway which observed in all electrophorograms in exposed time. Another degradation product, *i.e.* compound I, appears in the electrophorogram with the migration time of 16.67 min. In addition, the presence of compound II is not visible on this electrophorogram after ~10 days under the described conditions. All results were confirmed by recording of CVD solution’s color at different exposure times (Figure 2A). The reason of this phenomenon is the presence of aromatic residues and conjugated double bonds containing O and N in the structure which is usually associated with the ability of the molecule to absorb light (24).

**Figure 3:**
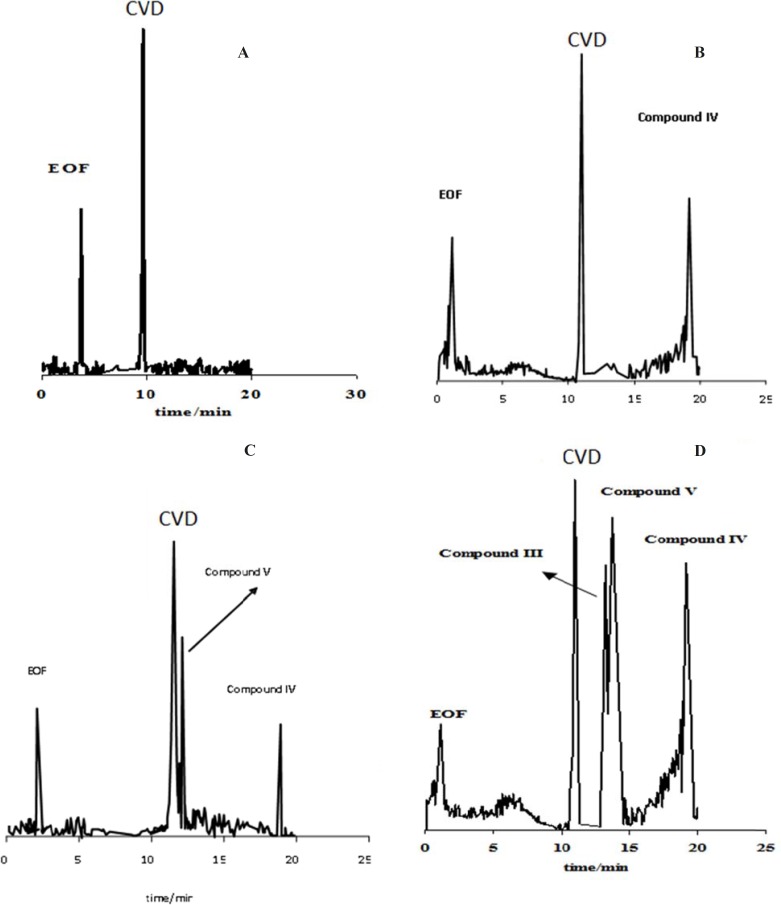
Electrophorogram of 1.14 mg/mL CVD for time=0 (immediately after preparation of CVD) (**A**) and (B-D) after 30-75 hours of acidic degradation in 0.1 M HCl. Experimental conditions: running buffer: acetate buffer (80 mM) in methanol: ethanol (65:35 v/v) with the apparent pH of 4.1, fused silica capillary (57 cm length and 75 µm I.D.); wavelength: 200-350 nm; applied voltage: 19 kV; temperature: 20 ºC.

For further study, the variations of CVD and degradation products absorbance were obtained versus time of exposure. Figure 2B shows that the absorbance of CVD and degradation products is proportional to the time from 0-30 day, pointing to the degradation of the CVD. As evident from the figure, the variations are more significant after ~10 days of exposure. Figure 2B indicates that CVD absorbance decreased with the time, signifying a degradation process of CVD. This result was confirmed by increasing on the degradation product absorbance. In addition, this result was verified by decreasing on the amount of CVD after photodegradation which indicated on Figure 3B. Also, Figure 3 shows that degradation products III and I were detected after ~10 and ~19 days with the migration times of 9.33 and 16.67 min, respectively.

On the bases of the experimental results, the proposed method is suitable for the simultaneous qualitative detection of CVD and its degradation products in pharmaceutical formulations. CVD can be exposed to daylight to obtain its degradation products. As the first degradation product, compound III was found by the proposed electrophoresis method.

The spectra of CVD and degradation products were acquired in an attempt to determine the structure of the degradation products. The degradation procedure of CVD was confirmed by FT-IR spectra (Scheme 1B). The oxidation of secondary alcoholic group of CVD to the corresponding ketones can be explained from its IR spectrum in which two peaks at 1730 and 1665 cm^-1^ were observed due to COCH_2_ and C=C groups, respectively, indicating the conversion of –CH-OH functionality to –CO- group (24).

On the bases of Table 1, in photodegradation conditions three new degradation products were detected which one of this products (*i.e*. compound II) is a neutral compound and degradation products I and III have 2 ionizable functional groups (C=O and N-H). Insomuch, molecular weight (MW) of CVD and photodegradation products are approximately equal, hence shorter migration time of degradation product III is expected because of existence of two ionizable groups (C=O and NH). In addition, degradation product I has lower ionization capability in pH=4.1 when compared with CVD. Hence, degradation product I has longer migration time than CVD. The direct correlation of degradation product structures and λ_max_ values with ionization groups and also relationship of these results with the migration time of the analytes are presented in Table 1. It can be seen that compound III has two ionizable groups and compound I has one NH as an ionizable group. Thus this makes the former degradation statistically more probable. This may be the reason of shorter migration time of degradation product III compared to CVD and other degradation products. It is clear that the presence of two ionizable groups facilitates the interpretation of CVD photodegradation. These results were confirmed by FT-IR spectra (Scheme 1B). It is observed that OH peak was decreased and two new peaks with wave numbers of 1732 (C=O) and 1665 (double band) were observed which related to mild photodegradation of CVD. These results were confirmed by other research groups or database (22-25). Galanopoulou *et al*. (24) indicted that part of CVD bearing amino and hydroxyl groups underwent a kind of modifying for constitution of C=O group. Also, Fields *et al*. (25) showed that the silght displacment of the aromanic protons could be contributed to the molecular modification such as conformation change, affecting long range intermoleqular intractions. In adddition, spectra database for organic compounds (SDBS) (26) confirmed these results. For elucidation of these results, UV-Visible absorption was used for evaluation of CE and FT-IR results. DAD can greatly simplify analysis of electrophoretic data. UV-DAD results were confirmed constitution of some degradation products with double band and C=O groups. Results obtained from UV-DAD investigation are given in Table 1. The UV spectra show a well-defined peak at 243 nm for CVD in the measuring wavelength range of 200-380 nm. On the other hand, after daylight exposure of CVD, two peaks were observed at 239 and 264 nm which is related to double band at contiguity of NH group (27, 28). Based on Table 1 results, a good accordance was obtained between CE methods with other previous reports (23-27). 

All of above results was discussed by investigation of partial surface area (PSA) of CVD and degradation products. In this study, PSA was used for evaluation of CVD and degradation product ionization groups. Its established molecular surface properties have been used to describe solvation and partial ionization processes for a long time. One of the most useful surface properties has been shown to be PSA, characterizing the polar part of the molecular surface, defined simply as the part of the surface corresponding to oxygen and nitrogen, and including also the hydrogen’s attached to these atoms (29). Scheme 1C indicated an extremely rapid method to obtain PSA which describe simply from the sum of contributions of polar fragments in a molecule. This figure shows that in photodegradation conditions the PSA of degradation product was vary as III>CVD> I or II which verify our results on CE proposed method. These results indicated that proposed methods for CVD degradation in acidic/ alkaline conditions is reliable.

*Force degradation of CVD*

**Figure 4:**
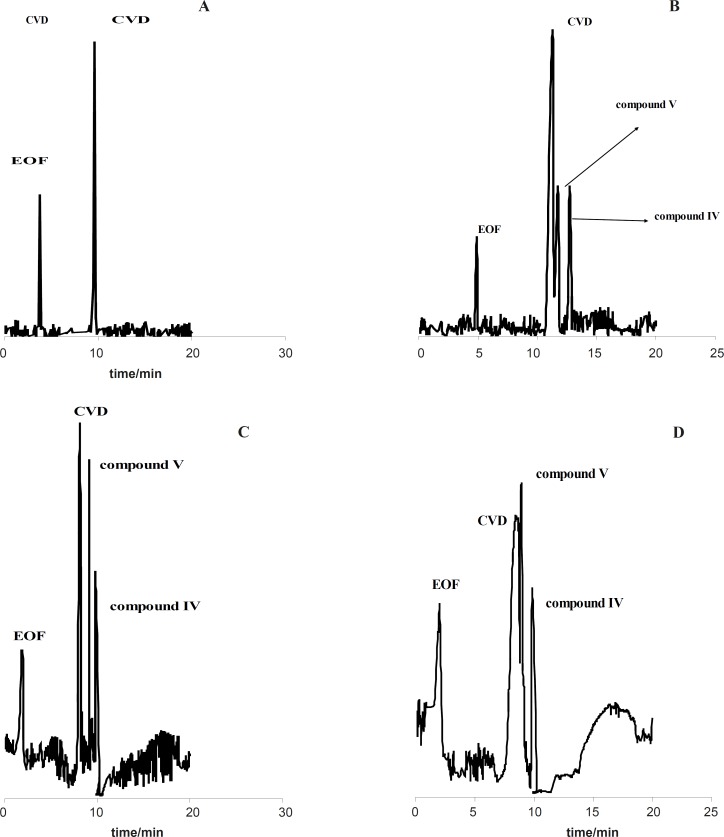
Electrophorogram of 1.14 mg/mL CVD for time=0 (immediately after preparation of CVD) (**A**) and (B-D) after 30-75 hour of alkaline degradation in 0.1 M NaOH. Experimental conditions: running buffer: acetate buffer (80 mM) in methanol: ethanol (65:35 v/v) with the apparent pH of 4.1, fused silica capillary (57 cm length and 75 µm I.D.); wavelength: 200-350 nm; applied voltage: 19 kV; temperature: 20 ºC.

The stress conditions carried out for forced degradation study comprised of acid hydrolysis (0.1 M HCl) and basic hydrolysis (0.1 M NaOH). The study period was 10-75 h at 60 ºC for acid and alkaline hydrolysis. The degradation samples were then cooled to room temperature. The forced degradation in acid and alkaline media was performed in the dark place to exclude possible photodegradation. Electrophorograms of CVD in two force degradation conditions (0.1 M HCl and 0.1 M NaOH at 60 ºC) in different time of reflux were illustrated in Figures 3 and 4. Figure 3 shows that degradation products V and IV were detected after 50 h and 30 h under acidic hydrolysis at 60 ºC with the migration times of 14.89 and 18.01 min, respectively. These results corroborate that degradation product IV was detected before degradation product V.

**Figure 5:**
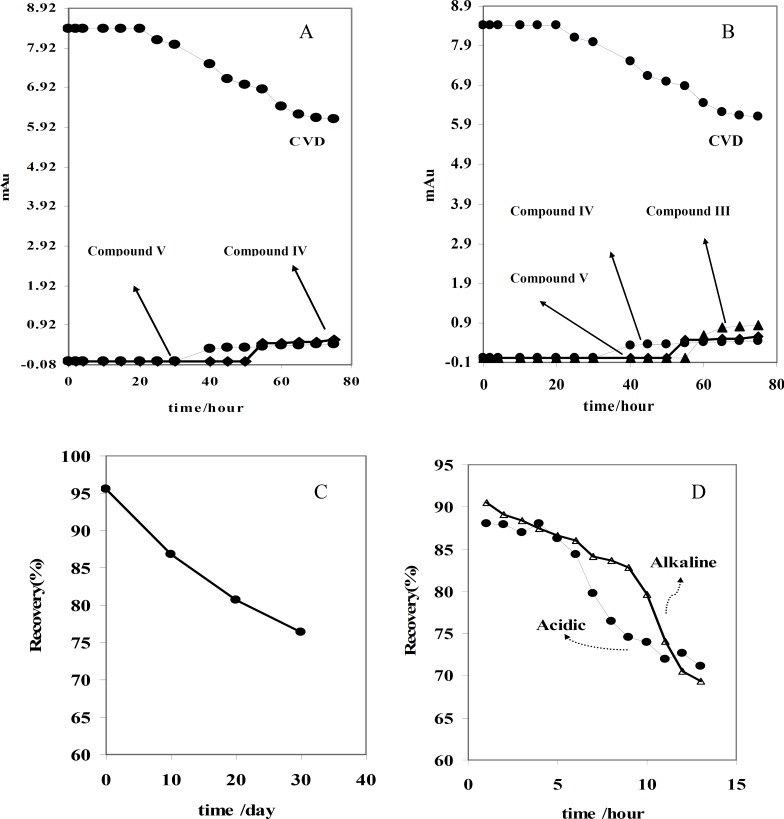
Electrophoretic behaviors of CVD and its degradation products in acidic (**A**) and alkaline (B) force degradation conditions. Recovery of CVD on photo (**C**) and acidic-alkaline (D) degradation conditions

**Scheme 2:**
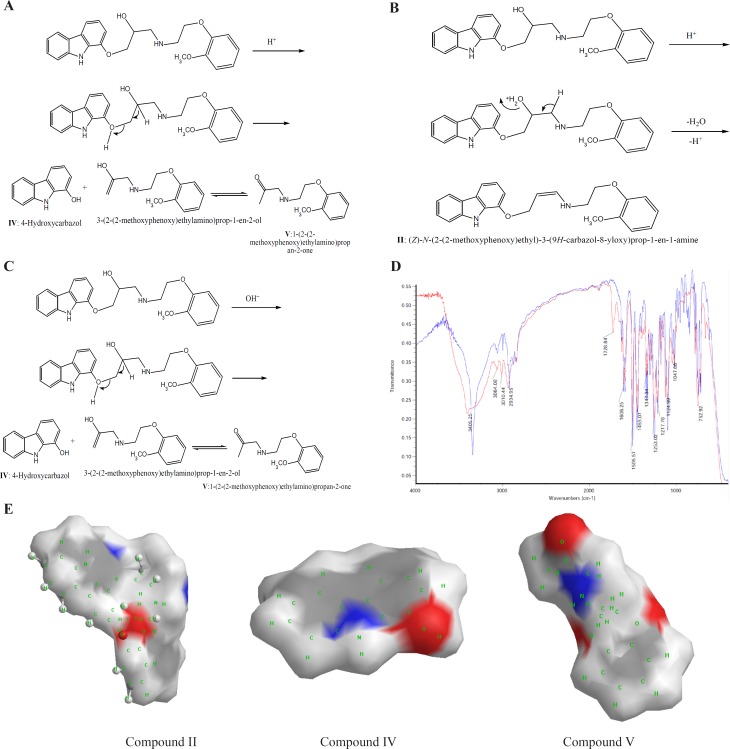
A) Degrading procedure of CVD on acidic force degradation conditions. B) Possible degrading procedure of CVD on acidic force degradation conditions for unknown product. C) Degrading procedure of CVD on alkaline force degradation conditions. D) FTIR spectrum of CVD at force degradation conditions. E) Partial surface area of CVD forces degradation products

Figure 5A indicates that CVD absorbance decreased with the time, indicating a degradation process of CVD. This result was sound by increasing on the degradation product absorbance. Considering these observations, the degradation mechanism in acidic hydrolysis could be proposed as Scheme 2A. This mechanism was confirmed by electromigration study of CVD in acidic force degradation conditions. Based on Table 1 results, degradation product V (MW=238 g/mol) with two ionizable have shorter migration time. Degradation product V has lower MW and two ionizable groups, therefore, it was observed at longer migration time in pH=4.1. All these results were confirmed by DAD data which was summarized in Table 1. After force degradation, UV spectra show two peaks at 284 and 236 nm which related to C=O and NH and NH group with lower mass than compound V. Considering these results with the electrophorograms, it is clarify this peak show appearance of compound V in this condition. Similar results were obtained in alkaline force degradation which was summarized in Table 1.

In addition; the presence of another unknown degradation product is observed after 50 h hydrolysis under the described conditions. This result was confirmed by increasing on the unknown product absorbance *vs.* time of hydrolysis (Figure 5A). Based on these observations, a new degradation mechanism in acidic hydrolysis was competing with prior degradation mechanism according to Scheme 2A&B. It is found that the amount of this new degradation product (*i.e.* compound II) is very low.

Alkaline force degradation was also performed and similar results were obtained as indicated in Figure 5B. The only difference is the absence of an unknown degradation product ((Z)-N-(2-(2-methoxyphenoxy) ethyl)-3-(9H-carbazol-8-yloxy) prop-1-en-1-amine). Based on these results, degradation mechanism in acidic hydrolysis described in Scheme 2C which was confirmed by FT-IR spectra (Scheme 2D). To provide more comprehensive information, Table 2 was compared with other available reports (22, 24) on CVD degradation products along with the findings of this work.

Scheme 2E illustrated that the PSA of acidic/alkaline degradations were varied as CVD>V>II>IV and CVD>V>IV, respectively. These results indicated that the proposed method for analysis of CVD degradation in acidic/ alkaline conditions is valid.

**Table 2:** Comparison of reported assays with proposed method for detection of CVD degradation products

**Method**	**Degradation products**	**Ref.**
RP-HPLC	((2RS)-1-(2-(2-methoxyphenoxy) ethyl) amino)-3-(9H-carbazol-4-yloxy) propan-2-ol	23
	4-hydroxycarbazole	
HPLC	N-[(2RS)-3-(9H-carbazol-4-yloxy)-2-hydroxypropyl]-N-[2-(2-methoxyphenoxy) ethyl] hydroxylamine	24
CE	**Photo degradation (mild conditions)**	This Work
	(E)-N-(2-(2-methoxyphenoxy)ethyl)-3-(9H-carbazol-8-yloxy)prop-2-en-1-amine	
	(Z)-N-(2-(2-methoxyphenoxy)ethyl)-3-(9H-carbazol-8-yloxy)prop-1-en-1-amine	
	1-(2-(2-methoxyphenoxy)ethylamino)-3-(9H-carbazol-8-yloxy)propan-2-one	
	**Force degradation in acid conditions**	
	4-Hydroxycarbazol	
	1-(2-(2-methoxyphenoxy)ethylamino)propan-2-one	
	(Z)-N-(2-(2-methoxyphenoxy)ethyl)-3-(9H-carbazol-8-yloxy)prop-1-en-1-amine	
	**Force degradation in alkaline conditions**	
	4-Hydroxycarbazol	
	1-(2-(2-methoxyphenoxy)ethylamino)propan-2-one	

*Validation of the method*

The described method was validated in terms of specificity, linearity, accuracy, precision, limits of detection (LOD) and quantification (LOQ), robustness and system suitability concerning ICH guidelines Q_2_A and Q_2_B (30, 31). The precision (% relative standard deviation) was expressed as the intra- and inter-day variations in the expected drug concentrations.

Linear calibration plots for CVD assay method was obtained over the calibration range nominal concentration, 240-700 µg/mL and the correlation coefficient obtained was 0.9986 (n = 10). The results exhibit that an excellent correlation existed between the peak area and concentration of the analyte. The plot of peak areas versus concentrations of CVD was found to be linear within the concentration ranges stated above. Details of validation data for CVD are summarized in Table 3.

**Table 3:** Some validation data of CVD from new CE method in prepared solutions with pH=4.1 acetate buffer (80 mM) on methanol/ethanol mixture (65:35%).

Linear range (μg/mL)	240-700
Slope	41.11
Intercept	106.08
R^2^	0.9986 (n = 10)
LOD a (μg/mL)	5.01
LOQ b (μg/mL)	11.40
RSD c (%)	3.1
Bias (%)	2.3
Repeatability	
Inter-day measured concentration	
Recovery (%)	96.53
RSD (%)	2.44
Bias (%)	2.14
Intra-day measured concentration	
Recovery (%)	95.11
RSD (%)	2.03
Bias (%)	2.00

a LOD is Limit of Detection

b LOQ is Limit of quantification

c RSD is Relative Standard Deviation

The ICH guideline (30) describes several approaches to determine the LOD and LOQ. These include visual evaluation, signal-to-noise ratio and the use of standard deviation of the response and the slope of the calibration curve. In the present study, the LOD and LOQ were based on the third approach and were calculated according to the 3r/s and 10r/s criteria, respectively; where r is the standard deviation of the peak areas and s is the slope of the corresponding calibration curve. The LOD and LOQ values of the proposed method are presented in Table 3.

The precision of the proposed method were assessed as repeatability and intermediate precision performing four replicate injections of the sample solutions at 100% level of the test concentration for CVD. The sample solutions were freshly prepared and analyzed daily and these experiments were repeated over a week period to evaluate day-to-day variability and with a different analyst to assess intermediate precision of the proposed method (Table 3).

Finally, accuracy of the proposed method was established by recovery experiments from spiked human serum samples. This study was employed by addition of 15 μg/mL of CVD or its related compounds (which was degraded by daylight, acidic and alkaline conditions) into the human serum. Results obtained from recovery studies are given in Table 4. Low recovery results related to degradation of CVD which supposed for this assay. Also it is found that recovery of CVD before degradation is approximately 95%. These results indicated that proposed assay procedure could be used for monitoring CVD degradation products in real samples (*i.e.* pharmaceutical solutions and serum samples). The results obtained for detection of CVD by the proposed CE method are compared with those of previously reported techniques (22, 23, 32-43). 

**Table 4:** Results obtained from recovery analysis of CVD in three conditions at human serum.

**Degradation Condition**	**Time**	**Added CVD to human Serum (μg/mL)**	**Measured CVD to human Serum ** **(μg/mL)**	**RSD (%)**	**Bias (%)**	**Recovery (%)**
Daylight (summer)	0 day	15	14.33	2.63	-2.11	96.5
	10 days		13.03	1.32	-2.00	86.9
	20 days		12.10	4.79	-3.20	80.7
	30 days		11.46	3.03	1.91	76.4
Acidic (0.1 M HCl)	10 h	15	13.21	5.17	0.49	88.1
	30 h		12.93	5.00	-1.67	86.2
	50 h		11.18	4.92	-3.00	74.5
	75 h		10.66	6.01	1.56	71.1
Alkaline (0.1 M NaOH)	10 h	15	13.57	6.00	1.00	90.5
	30 h		13.00	6.00	0.89	86.7
	50 h		12.42	3.77	-0.77	82.8
	75 h		10.41	5.12	2.96	69.4

CE methods have some of difference with other reports such as: analysis times, sensitivity, selectivity and dynamic range. In terms of analysis time, CE offers substantial advantages compared to other reported methods. In the present method, the analysis time required for degradation products separation was about 20 min whereas for the HPLC assay, an analysis time of 34 min was necessary (25). While the HPLC required an equilibration time of about 1 h prior to analysis. The low consumption of solvents and buffer additives is also a major advantage in CE. In this study, only 10 mL of buffer was sufficient to perform about 20 determinations. One of the principal limitations of CE is its sensitivity. Due to the extremely small diameter of the capillary, volumes of nanoliters of the sample solutions are typically injected. For the same reason, the light path at the on-capillary detector window is very short and consequently the mass of the analyte in the detection window is very low, reducing the signal produced by the sample. For this reason, the proposed CE method for CVD in serum possesses an LOD of 5.01 μg/mL whereas HPLC (22, 23), voltametry (34), UV (37) and spectrofluorimetry (43) provided LODs of 0.005 µg/mL, 1.7 µg/ mL, 0.7 µg/mL and 1.90 ng/mL, respectively. Direct injection of the serum samples to the capillary is a great advantage for CE methods, especially for routine analysis of large number of serum samples in the clinical laboratories. In terms of injection volumes, the injection volume of 18 nL was used in the proposed CE method. Based on Table 1, it can be seen that the selectivity at the detector was actually higher for CE compared with HPLC and UV. On the other hand, the sensitivity limitations of CE are related to the small quantities injected and consequently dependent on the concentration of the starting material. This is particularly significant in pharmacokinetic studies where drug concentrations are often extremely low, an example being CVD. In addition, on some of developed HPLC methods it is observed that some of degradation peaks were overlapped which was not observed in CE method. Also from these studies it would appear that CE detected a further degradation product which undetectable by HPLC. In addition, in the spectrofluorimetry method (40), high sensitivity is generally expected. However, problems of selectivity can occur in multi-component analyses because of the overlap of their spectra that could be observed.

Results indicated that the proposed CE method compares well with HPLC, UV and electrochemical methods and can be used for the determination of CVD in human serum. Although limits of detections are lower with HPLC and UV, the CE assay offers the advantage of faster analysis times and low consumption of solvents. In addition, due to different separation mechanism of CE method, it provides an orthogonal analysis in combination with HPLC method to detect more degradation products (21). 

The degradation of sample solutions was tested by CE over a period of 30 days and 75 hour under photo and force degradation conditions, respectively. The freshly prepared solutions at room temperature and degradation conditions were analyzed by the proposed CE method. The concentrations of CVD in the above conditions were calculated and compared to the freshly prepared samples (Figures 5 C and D). From these results it is found that CVD is not stable at daylight and force degradation conditions, indicating the possibility of CVD degradation.

## Conclusions

A simple and sensitive CE assay method was proposed for the monitoring of CVD degradation. The results indicated that CE method is appropriate for simultaneous detection of CVD and its degradation products in the serum samples. Therefore, this method is suitable for the chemical purity determination, as well as for stability control of CVD. In addition, with the CE method, three new degradation products of CVD were detected.
